# Advances in CAR-T Cell Therapy in Head and Neck Squamous Cell Carcinoma

**DOI:** 10.3390/jcm12062173

**Published:** 2023-03-10

**Authors:** Han-Qi Wang, Ruxing Fu, Qi-Wen Man, Guang Yang, Bing Liu, Lin-Lin Bu

**Affiliations:** 1 The State Key Laboratory Breeding Base of Basic Science of Stomatology (Hubei-MOST) & Key Laboratory of Oral Biomedicine Ministry of Education, School & Hospital of Stomatology, Wuhan University, Wuhan 430072, China; 2 Department of Oral & Maxillofacial Head Neck Oncology, School & Hospital of Stomatology, Wuhan University, Wuhan 430072, China; 3 Department of Materials Science and Engineering, University of California, Los Angeles, CA 92093, USA; 4 Department of Biomedical Engineering, College of Life Science and Technology, Huazhong University of Science and Technology, Wuhan 430074, China

**Keywords:** CAR-T cell, cancer therapy, immunotherapy, solid tumor, HNSCC

## Abstract

Surgery with the assistance of conventional radiotherapy, chemotherapy and immunotherapy is the basis for head and neck squamous cell carcinoma (HNSCC) treatment. However, with these treatment modalities, the recurrence and metastasis of tumors remain at a high level. Increasingly, the evidence indicates an excellent anti-tumor effect of chimeric antigen receptor T (CAR-T) cells in hematological malignancy treatment, and this novel immunotherapy has attracted researchers’ attention in HNSCC treatment. Although several clinical trials have been conducted, the weak anti-tumor effect and the side effects of CAR-T cell therapy against HNSCC are barriers to clinical translation. The limited choices of targeting proteins, the barriers of CAR-T cell infiltration into targeted tumors and short survival time in vivo should be solved. In this review, we introduce barriers of CAR-T cell therapy in HNSCC. The limitations and current promising strategies to overcome barriers in solid tumors, as well as the applications for HNSCC treatment, are covered. The perspectives of CAR-T cell therapy in future HNSCC treatment are also discussed.

## 1. Background

Head and neck squamous cell carcinomas (HNSCCs) are malignant lesions of the mucosal epithelium from the oral cavity, pharynx and larynx and are regarded as the most common malignancies in head and neck cancer, accounting for 90%. As the sixth most common malignancy worldwide, there were an estimated 880,000 new cases and 440,000 deaths from HNSCC worldwide in 2020 [1,2]. The high-risk factors of HNSCC present a regional difference. In European countries, increasing HPV infection contributes to the incidence of HNSCC, while addiction to alcohol and tobacco and the consumption of areca are the common high-risk factors in south Asian countries [3,4,5]. Nowadays, the treatment modalities of HNSCC are dependent on the TNM staging system and the multidisciplinary team working on the case, comprising surgery with the supplementation of radiotherapy and chemotherapy, which remains the basic modality. Although the treatment modalities for HNSCC have been continuously improved, the five-year survival rate of patients has remained at approximately 50–66% in the last 40 years [1,6]. Moreover, due to the special lesion site of HNSCC, surgery can destruct the maxillofacial region, seriously affect facets of daily life such as chewing, speaking and sociality, reduce the quality of life and increase the psychological burden of patients. It has been reported that the suicide rate amongst HNSCC survivors in the USA (63.4 of 100,000 person-year) is the second highest among all kinds of cancer following pancreatic cancer (86.4 of 100,000 person-year), and is three times higher than the suicide rate of the general population [7]. Therefore, improving the anti-tumor efficacy of treatment and the life quality of patients has become the current research focus.

**Figure 1:**
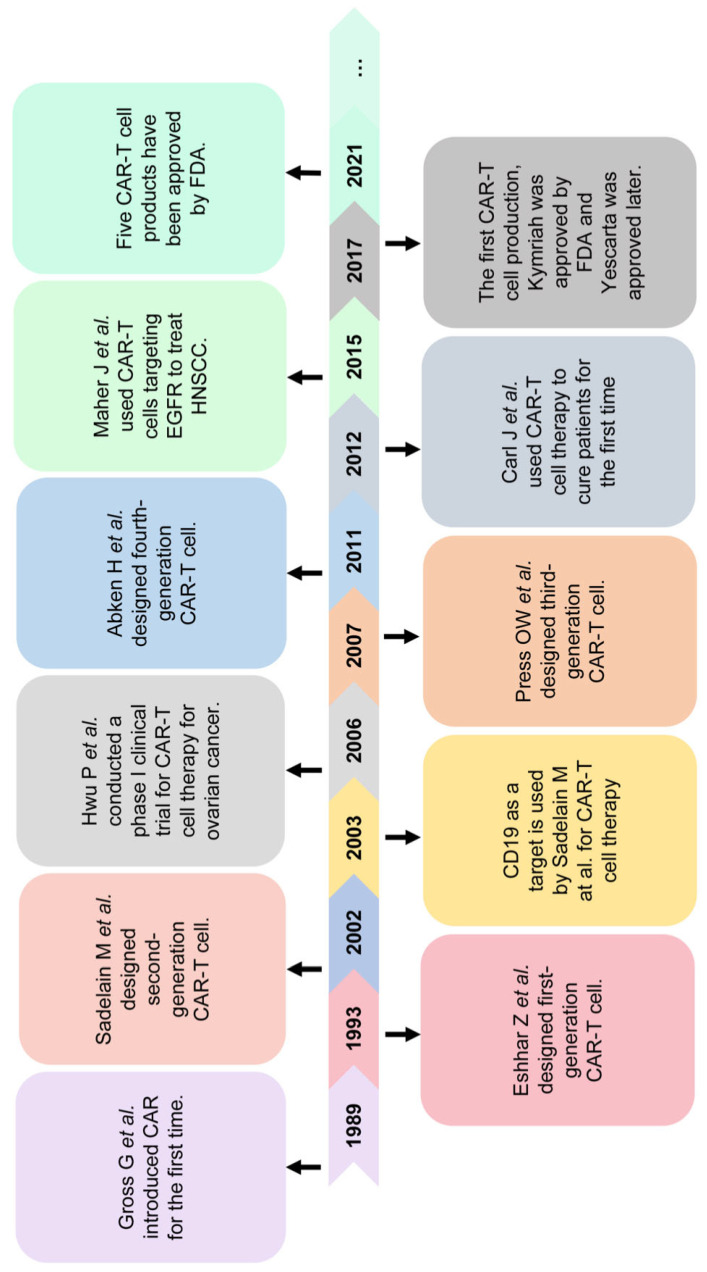
Timeline of chimeric antigen receptor T cell therapy [11,13,14,15,16,17]. In 1989, Gross et al. first reported the chimeric antigen receptor T cell which can specifically recognize tumor surface antigens in a non-MHC-restricted manner. In the following years, a total of 4 generations of CAR-T cells were developed and reported. In 2012, Carl June and co-workers successfully cured a patient with acute B-cell lymphoblastic leukemia using CAR-T cell therapy. Now, the patient has survived tumor-free for 9 years. CAR-T cell therapy for solid tumors including HNSCC has also been reported in recent years. Maher J and co-workers demonstrated EGFR as being one of the targets of HNSCC therapy. In 2017, two CAR-T cell productions targeting CD19, Kymriah and Yescarta, were approved by the FDA for the treatment of hematological malignancies. Subsequently, three more CAR-T cell productions were approved by the FDA.

Immunotherapies—including tumor vaccines, adoptive immune cell therapy and immune-checkpoint-blocking—have shown great potential in recent preclinical studies and clinical trials [8,9,10]. Chimeric antigen receptor T (CAR-T) cell therapy, a novel immunotherapy, was first put forward in the 1980s and shows excellent anti-tumor efficacy in hematological malignancies. In CAR-T cell therapy, T cells derived from patients are genetically engineered to express antibodies that can specifically recognize tumor antigens in a non-major histocompatibility complex (MHC)-restricted manner (Figure 1) [11,12]. To date, five CAR-T cell products approved by the FDA have been used for the treatment of hematological malignancies. The excellent performance of CAR-T cell therapy in the treatment of hematological malignancies, and its anti-tumor effect in solid tumors, including HNSCC, have attracted the focus of studies. This review introduces the current development, barriers and solutions of CAR-T cell therapy. We also summarize the advances in CAR-T cell therapy for the treatment of HNSCC and discuss the opportunities and challenges of CAR-T cell therapy in HNSCC.

## 2. Brief Introduction to CAR-T Cell in Cancer Treatment

**Figure 2:**
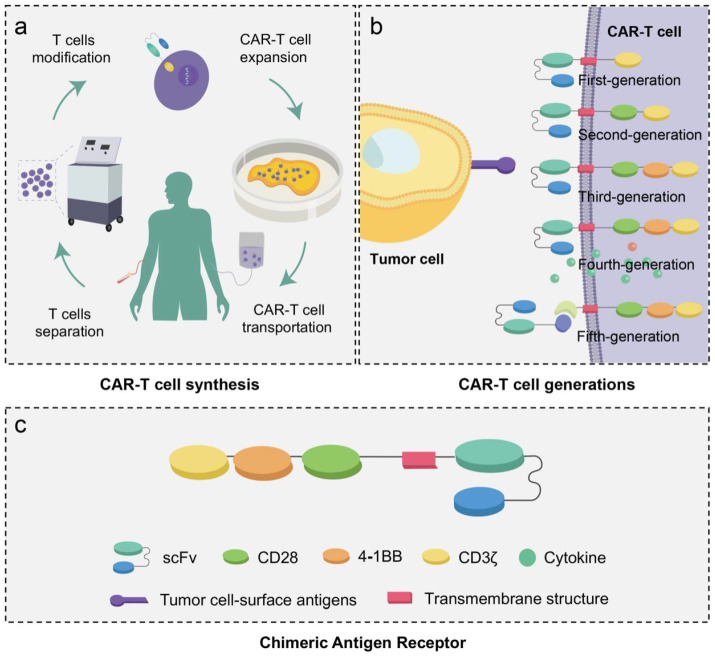
Schematic of CAR-T cell synthesis and five generations of the CAR-T cell. (**a**) Cells are separated and collected via leukapheresis, monocyte elutriation and T-cell selection. The isolated T cells are transduced to express CAR proteins through genetic engineering. After transgene delivery, CAR-T cells are expended in vitro and transported into the patients. (**b**,**c**) CAR is composed of a single-chain variable fragment, transmembrane domain and signal domain. The signal domain of the first-generation CAR-T cell is typically composed of the CD3ζ signal chain. In second- or third-generation CAR-T cells, the structure of the signal domain contains co-stimulatory domains such as cluster of differentiation 28 and/or 4-1BB. The fourth-generation CAR-T cell is engineered to be equipped with the nuclear factor and express cytokines. The structure of CAR-T cells is still improving, and the development of a fifth-generation CAR-T cell is inevitable.

The synthesis of CAR-T cells modifies the patient’s autologous T cells through genetic engineering technology in vitro. The process includes three main steps: (1) the T cells of patients are separated and collected via leukapheresis, monocyte elutriation and T cell selection; (2) the isolated T cells are activated and transduced by viral vectors encoding specific CAR genes and (3) after transgene delivery, the CAR-T cells are expended in vitro and transported into the patient’s body to achieve the targeting and killing of tumor cells (Figure 2a) [18]. Due to the low expression of MHC molecules, tumor cells have the characteristic of low immunogenicity. To enhance the effect of tumor cell recognition, compared to traditional immunotherapy, CAR-T cells are designed to recognize tumor cells in an MHC-independent manner. In this way, CAR-T cell therapy can reduce the impact of immune escape caused by the low immunogenicity of tumor cells. Moreover, CARs have the advantage of strong controllability and can increase T cell activation [19].

The structure of CAR-T cells contains T cells and CARs that can specifically recognize tumor cells. The extracellular domain of CARs is an antigen-binding single-chain variable fragment (scFv) composed of a variable light chain (V_L_) and variable heavy chain (V_H_). Connected to a transmembrane structure, the scFv is derived from antibody proteins that can specifically recognize tumor-associated antigens. The intercellular domain of CARs in T cells is a signal domain composed of a CD3ζ signal chain and co-stimulation domains, which can mediate T cell activation in a T-cell-receptor-independent manner [12]. According to different intracellular domains, CAR-T cells are divided into four generations. Recently, researchers have reported on fourth-generation CAR-T cells (T cells redirected for universal cytokine killing, TRUCK), which are engineered to release pro-inflammatory cytokines, including interleukin (IL)-12, IL-8, and IL-9, to increase the activation of CAR-T cells and other innate immune cells [18,20,21]. Furthermore, due to the high cost of CAR-T cell therapy and the complicated synthesis process of CAR-T cells (the prices of Kymriah and Yescarta are about USD 473,000 and USD 373,000, respectively) [22], the existence of “fifth-generation” CAR-T cells has been reported (universal CAR-T, UniCAR-T). This CAR system is split into two parts (the intracellular signal domain and the extracellular domain scFv) to identify more target proteins and improve the flexibility of CAR-T cells in treating different cancers (Figure 2b) [23].

In addition to efficacy, safety is an aspect that cannot be ignored when evaluating a new treatment modality. Due to its “on-target, off-tumor” toxicity, CAR-T cell therapy can cause a series of immunopathological reactions, including fever, hypotension, hypoxia, neurotoxicity, skin toxicity, gastrointestinal toxicity and multiple organ damage. It can even lead to death in severe cases, caused by cytokine release syndrome (CRS; an acute systemic inflammatory syndrome) or tumor lysis syndrome [24,25,26]. Because of these adverse events, the clinical application of CAR-T is limited. Therefore, in addition to enhancing the anti-tumor efficacy, the method of eliminating the complications of CAR-T cell therapy is also a big challenge in the clinical translation of CAR-T cell therapy.

An appropriately selected CAR target protein is of great significance in enhancing anti-tumor efficacy and reducing the complications of CAR-T cell therapy. A variety of proteins overexpressed in tumor cells are selected as the protein targets. The epidermal growth factor receptor (EGFR) is overexpressed in lung cancer, and EGFR inhibitors have been reported as the first targeted drugs for lung cancer treatment. Its variant III (EGFRvIII) is expressed in 30%–40% of glioblastomas (GBMs) [27,28]. Clinical trials of EGFR and EGFRvIII-targeted CAR-T cells in treating metastatic colorectal cancer, metastatic pancreatic cancer, recurrent GBM, brain cancer and glioblastomas have been conducted [29,30,31]. Human epidermal growth factor receptor 2 (HER2, ErbB2) has been demonstrated to overexpress in solid tumors, including GBM, breast cancer, ovarian cancer and digestive system malignancies [32,33,34,35]. There are various clinical trials using HER2-targeted CAR-T cells for the treatment of HER2-positive sarcoma, progressive GBM, advanced biliary tract cancers and pancreatic cancers [36,37,38]. The IL-13 receptor α2 (IL13Rα2) has been confirmed to be overexpressed in almost 75% of GBMs, which are associated with tumor invasion and poor survival rates [39]. Clinical trials of IL13Rα2-targeted CAR-T cells for the treatment of recurrent GBM have been reported [40,41]. 

## 3. Application of CAR-T Cell Therapy in Head and Neck Squamous Cell Carcinomas

CAR-T cell therapy in solid tumors has been demonstrated to be a promising therapeutic strategy. As HNSCC is a malignant tumor that seriously affects patients’ health and quality of life, CAR-T cell therapy of HNSCC is also the focus of current research. Nowadays, studies on CAR-T cells for treating HNSCC (Table 1) are still focused on preclinical research, while the transformation of preclinical studies into clinical trials is still not optimistic. 

### 3.1. Preclinical Research of CAR-T Cell Therapy against HNSCC

HER2-targeted CAR-T cells have been present in preclinical research for a long time for the treatment of solid tumors. It has been demonstrated that HER2 can be detected in 0–47% of HNSCC tissues, and the overexpression of HER2 is associated with a worse prognosis of patients with HNSCC [57]. Due to the relationship between the HER2 expression level and HNSCC progression, Warren et al. developed HER2-specific CAR-T cells for the treatment of HNSCC. The results showed that anti-HER CAR-T cells achieved a 56% decrease in tumor size, suggesting HER2 could be a potential target in CAR-T cell therapy against HER-positive HNSCC [58]. 

EGFR is overexpressed in hypopharyngeal carcinoma, which constitutes approximately 5% of HNSCC. Dong et al. developed EGFR-targeted CAR-T cells to limit the growth of an EGFR-positive hypopharyngeal carcinoma cell line. The results showed that the cytokine secretion and lysis rates of target cells were significantly enhanced after the co-culture of target cells and CAR-T cells [43].

CD70, serving as a tumor necrosis factor ligand, is highly expressed in solid tumors, including HNSCC. The overexpression of CD70 is related to a reduction in CD8^+^ T cells, which can induce immunosuppressive TME [59]. Park et al. analyzed nine proteins that were highly expressed in HNSCC cells as being potential CAR-T cell targets and demonstrated that anti-CD70 CAR-T cells can effectively eliminate HNSCC cells compared to the no-treatment group [44].

The expression of mucin 1 (MUC1) and its associated epitopes have been detected in HNSCC and are regarded as possible prognostic markers. Their overexpression has been reported to be associated with an adverse tumor stage and radioresistance, so targeting MUC1 has become a promising strategy for HNSCC treatment [60]. Mei et al. selected MUC1 as the target of CAR-T cells and engineered a fourth generation of CAR-T cells secreting IL-22 for the treatment of MUC1-positive HNSCC tumors in a xenograft mice model. It has been confirmed that MUC1-targeting IL22-CAR-T cells have great efficacy in inhibiting HNSCC growth in vivo [45].

Haist et al. reported that CD44v6-targeting CAR-T cells play an important role in killing CD44v6-positive HNSCC cell lines, including UM-14C, and their cytotoxicity depends on the tumor-associated CD44v6 expression levels. Almost 100% of tumor cells were killed after CAR-T cell therapy [46].

B7-H3, an immune checkpoint protein in the B7 family of cell surface molecules, is reported to play a role in impairing the T cell response, promoting the proliferation of immunosuppressive cells and enhancing the progression of tumors [61]. A preclinical study conducted by Scribner et al. showed that the single administration of MGC018, an antibody of B7-H3, achieved a 98% reduction in head and neck xenografts, indicating that the targeting of B7-H3 in CAR-T cell therapy exerts an anti-tumor effect in HNSCC treatment [47].

### 3.2. Clinical Trials of CAR-T Cell Therapy against HNSCC

The results of clinical trials on CAR-T cells for the treatment of HNSCC on the professional clinical trial registration website are not abundant. The safety and cytotoxic efficacy of an injection of CAR-T cells targeting ERBb (T1E28z) for HNSCC patients have been verified. The results showed that among the 12 evaluated patients, the overall disease control rate was 69% after intratumoral application [50]. Daniel Wang et al. published a recruitment notice for a phase I clinical trial of HNSCC treatment on the website in 2018 to explore the safety and cytotoxicity of HER2-targeting CAR-T cells combined with an intra-tumor injection of binary oncolytic adenovirus CAdVEC against HER2-positive tumors, including HNSCC. They also studied whether these CAR-T cells could survive in the blood. However, CAR-T cells targeting the epithelial cell adhesion molecule (EpCAM), natural killer group 2 member D ligand (NKG2DL) and latent membrane protein (LMP1) remain in clinical trials to verify their safety and efficacy for the treatment of nasopharyngeal carcinoma.

### 3.3. Potential Targets for CAR-T Cell Therapy against HNSCC

Looking for new targets of HNSCC to increase the specificity and reduce the side effects of CAR-T cells can promote the transformation of CAR-T cell therapy into clinical applications. The selection of CAR-T cell targets for the treatment of HNSCC is usually based on the following two prerequisites: (1) CAR-T cell target proteins that have been reported in the treatment of solid tumors, such as FAP and GD2; (2) proteins that are highly expressed on the surface of HNSCC cells and are rarely expressed in normal tissues. In addition to CD70, introduced by Park et al., MICA, MICB, MAGEA-4, FAP and β4GALNT1 are regarded as potential target proteins, considering the significant overexpression of these proteins on the surface of HNSCC cells compared to normal tissues [44]. This study paved the way for new targets for CAR-T cell selection in HNSCC. In addition to selecting tumor-specific surface antigens of HNSCC cells, researchers are also paying attention to the antigens of other solid tumors to find safe and feasible CAR-T cell targets.

## 4. Barriers of CAR-T Cell Therapy in HNSCC and Potential Solutions

Even though the earliest clinical trial of CAR-T cell therapy was conducted in solid tumors, thus far, the five FDA-approved CAR-T cell products are all in blood diseases. The reasons for the slow advancement in CAR-T cells for the treatment of HNSCC are associated with the barriers formed by the unique tumor microenvironment (TME) of solid tumors, and are as follows: (1) physical barriers, including compact tumor structures, tumor stromal cells, etc., (2) physiochemical barriers, including the downregulation of cytokines and acidic, hypoxia and low-nutrient environments and (3) pathological barriers, including immunosuppressive mechanisms, immune checkpoints, tumor antigen loss and heterogeneity, etc. These barriers restrict the infiltration of CAR-T cells into HNSCC, influence the specificity of HNSCC cell targeting and decrease the proliferation and anti-tumor effect of CAR-T cells [62,63,64]. Strategies to overcome these three barriers in solid tumors are regarded as the key point of accelerating the clinical translation of CAR-T cell therapy. Therefore, here, we introduce the barriers of tumor-site infiltration, anti-tumor cytotoxicity and the safety issues of CAR-T cells for HNSCC treatment.

### 4.1. Infiltration Barriers of CAR-T Cells in HNSCC

The prerequisite for CAR-T cells to exhibit cytotoxicity is transportation to and accumulation in the tumor site. However, compared to hematological tumors, there are many barriers blocking CAR-T cell infiltration into HNSCC, including angiogenesis, dense fibrous structure in the tumor extracellular matrix, downregulation of chemokine expression and mismatch between the chemokine receptors of T cells and the chemokines of tumor cells [62,65,66,67]. To deal with these transportation barriers, researchers have proposed the following several strategies.

#### 4.1.1. Delivery Techniques

**Figure 3:**
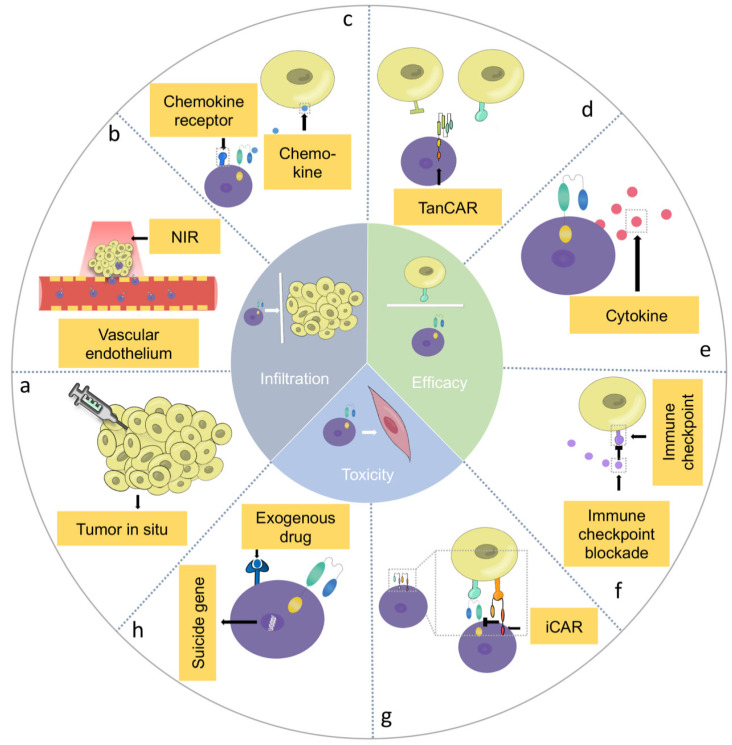
Methods to overcome the barriers of CAR-T cells in the treatment of HNSCC. There are three aspects in overcoming the barriers including promoting CAR-T cell infiltration to the solid tumor (**a**–**c**), activation (**d**–**f**) and reducing cytotoxicity in normal cells (**g**,**h**). (**a**) Intratumoral injection of CAR-T cell. (**b**) Photothermal pre-treatment followed by CAR-T cell therapy. (**c**) Expression of chemokine receptor through genetic engineering. (**d**) Tandem CAR-T cell targeting multiply proteins. (**f**) Secretion of immune-checkpoint-blocking proteins through genetic engineering. (**g**) iCAR-T cell. (**h**) Expression of suicide genes. (NIR: near infrared; TanCAR-T: tandem CAR-T cells; iCAR: inhibitory CAR.

Delivery techniques are reformed for the delivery of CAR-T cells into HNSCC. In addition to delivering CAR-T cells through intravenous injection, many studies have reported a direct intratumoral injection of CAR-T cells to treat a variety of solid tumors, including HNSCC, liver cancer, glioblastoma and breast cancer, effectively increasing the accumulation of CAR-T cells at the tumor site while reducing their cytotoxicity to normal tissue cells (Figure 3a) [68,69,70,71]. The administration of T1E28z CAR-T cells against ErbB^+^ HNSCC in a mouse model via the intratumoral route presented a more significant anti-tumor effect compared to the administration of CAR-T cells via the intravenous route. Moreover, no remarkable CRS or weight loss were observed among the mice treated via intratumoral CAR-T cell injection compared to those treated via intraperitoneal injection, indicating the safety of the administration of CAR-T cells using the intratumoral route [71].

Moreover, novel drug delivery systems can also play a significant role in improving CAR-T cell migration. It has been demonstrated that metal-based scaffolds and biopolymer scaffolds implanted into the tumor site can play an important role in promoting the infiltration, survival and efficacy of CAR-T cells. Stephan et al. harbored ovarian-cancer-specific T cells (NKG2D-targeted CAR-T cells) and T cell stimulants into bioengineered polymer matrices and used them for tumor treatment by situating implants near the tumor. On the 40th day after treatment with bioactive polymer scaffolds delivering CAR-T cells, the survival rate of the mice was 100%, while no mouse survived in the control group (*p* < 0.0001, *n* = 10) [72]. Gu et al. developed a new post-surgery local drug delivery system constituted by a biodegradable hydrogel reservoir for the treatment of a melanoma xenograft mouse model. CAR-T cells, human platelets conjugated with anti-programmed death-ligand-1-blocking antibody (aPDL1) and IL-15 were encapsulated into the hydrogel reservoir (CAR-T-P–aPDL1@gel) and released into the inflammatory TME of the surgical bed to inhibit the tumor growth and prolong the survival time of the treated mice [73]. Coon et al. developed a nitinol thin film to deliver CAR-T cells for the treatment of non-resectable ovarian cancer. The study showed that the survival rate of the mice was 70% on the 120th day after T cell transfer, compared to a 55-day median survival time in the local injection group (*p* < 0.0001, *n* = 10) [74].

#### 4.1.2. Combination Therapy

The combination of CAR-T cell therapy with other treatments was developed to break the physical barriers derived from vascular endothelial cells and induce the infiltration of CAR-T cells (Figure 3b). Chen et al. designed a novel way to overcome the barriers and enhance the efficacy of CAR-T cells in combination with photothermal therapy (PTT). After two-minute near infrared laser irradiation, the temperature of the tumor site was raised to 44 °C, which can kill tumor cells directly, reduce the physical barrier of tumor vasculature, promote the infiltration of CAR-T cells and increase the release of cytokines, including IL-12 and IFN-γ. In the melanoma xenograft model, after 20-day CAR-T cell treatment combined with PTT, the tumor volume was less than 100 mm^3^, demonstrating a significant inhibition of tumor growth [75].

#### 4.1.3. Genetically Engineered Multi-Functional CAR-T Cells

CAR-T cells are reported to express certain enzymes or chemokines through genetic engineering to enhance the infiltration into HNSCC. The expression of chemokine receptors that can identify the chemokines expressed by tumor cells on CAR-T cells via genetic engineering is regarded as one of the ways to increase the targeted infiltration of CAR-T cells. Research conducted by Jin et al. showed that in a glioblastoma xenograft model, the expression of the C-X-C motif chemokine receptor (CXCR) 1 and CXCR2 could significantly enhance the infiltration of CAR-T cells, and a large quantity of CAR-T cells could migrate to the tumor site two days after injection. Moreover, the survival rate of the mice after 80 days of injection was 100%, compared to 50% in the control group (*p* < 0.05) (Figure 3c) [20].

In addition to chemokines, some enzymes have been demonstrated to be effective in improving CAR-T cell migration. Heparanase (HPSE) is an endoglycosidase and has the ability to degrade heparan–sulphate proteoglycans, the main component of the tumor extracellular matrix, to remodel the extracellular matrix and promote the aggressiveness and chemoresistance of tumors [76]. Caruana et al. designed a novel CAR-T cell expressing HPSE through genetic engineering to improve its capability to degrade the extracellular matrix, increasing the infiltration and anti-tumor activity of CAR-T cells [77].

### 4.2. Efficacy Barriers of CAR-T Cells in HNSCC

#### 4.2.1. Genetic Heterogeneity in HNSCC

In addition to infiltration barriers, the genetic heterogeneity in HNSCC and the limitation of specific target selection can also cause tumor immune escape in traditional CAR-T cell therapy. In cancer treatment, researchers always screen specific molecular alterations from patients to achieve targeted therapy. However, patients with HNSCC do not greatly benefit from targeted therapy in clinical applications due to the lack of reliable specific biomarkers in HNSCC therapeutic options [78]. Therefore, strategies to enhance the targeting ability and achieve a controllable anti-tumor efficacy of CAR-T cells to HNSCC cells are regarded as novel ways to overcome the barriers of genetic heterogeneity in CAR-T cell therapy in HNSCC treatment.

To increase the T cell response and the lysis rate of tumor cells, researchers have designed multi-antigen-targeting CAR-T cells for the treatment of solid tumors. Li et al. used tandem CAR-T cells (TanCAR-T) targeting the HER2 and IL13Rα2 proteins for the treatment of glioblastomas and proved that they could address the problem of tumor antigen escape and increase the anti-tumor effect (Figure 3d) [79]. Cho et al. introduced a “split, universal, and programmable” (SUPRA) CAR system, which can change the targets of CAR-T cells without genetically reprogramming the CAR-T cells to achieve a controllable anti-tumor efficacy. In addition to the considerable anti-tumor cytotoxic efficacy, the SUPRA CAR system can achieve controllable CAR-T cell activation through the “switch” of T cells, the selection of target cells, the control of different subtypes of T cells and the control of different signaling domains to reduce the occurrence of CRS and enhance its safety and anti-tumor efficacy [80].

Some combination therapies and multi-function CAR-T cells have also been put forward. Park et al. improved the efficacy of CD19-targeting CAR-T cell therapy in solid tumors by combining CAR-T cells with oncolytic vaccinia viruses (OVs). OVs can preferentially replicate in tumor cells and potentiate the immune response of T cells and innate immune cells. Moreover, through genetic engineering, OVs can express a specific antigen on the tumor cell surface that can be recognized by CAR-T cells to enhance the anti-tumor effect of CAR-T cells [81]. In their study, the oncolytic vaccinia virus coded for the truncated nonsignaling variant of CD19 (OV19t) worked as a vehicle to selectively infect tumor cells and express CD19 on the surface of tumor cells, and then these infected tumor cells could be targeted by CAR-T cells [82]. Kagoya et al. designed a CAR-T cell encoding the intracellular domain from IL-2Rβ and STAT3-binding YXXQ motif. In an NALM6 cell xenograft mouse model, the activation of the JAK-STAT3/5 pathway was promoted, which enhanced the proliferation of CAR-T cells and significantly prolonged the survival of mice [83].

#### 4.2.2. Immunosuppressive Tumor Microenvironment in HNSCC

The acidic, hypoxia, low-nutrient TME and the overexpression of immune checkpoints associated with immune escape in HNSCC also have an adverse effect on CAR-T cells by inhibiting the ability of immune cells to target tumor cells [84,85,86]. The therapeutic efficacy of CAR-T cells alone has been confirmed to be insufficient in treating HNSCC, so a new way of improving CAR-T cells has been reported. Rosewell et al. pre-treated HNSCC with engineered binary oncolytic adenovirus (CAd) that can express programmed cell death-ligand 1 (PD-L1) blockade antibodies and cytokines, thereby improving the anti-tumor effect of HER2-targeting CAR-T cells [42].

Moreover, genetically engineered CAR-T cells that can release cytokines to alter the immunosuppressive TME have become a new option to increase the activity of CAR-T cells in some solid tumors (Figure 3e). Yeku et al. designed an armored CAR-T cell that can secrete IL-12. They demonstrated that IL-12 secretion can overcome immunosuppression, promote the proliferation and cytotoxicity of CAR-T cells and inhibit their apoptosis in ovarian cancer [87]. CAR-T cells releasing IL-23, IL-18 and IL-7 through the genetic engineering or preconditioning of CAR-T cells with IL-15 and IL-7 have also been demonstrated to achieve a remarkable cytotoxic effect against solid tumors [88,89,90,91].

In addition to CAR-T cells engineered to release cytokines, developing CAR-T cells that can express immune-checkpoint-blocking proteins is another way to increase the inhibitory effect of CAR-T cell therapy on tumors. Huang et al. engineered B7-H3-targeting CAR-T cells to co-express PD-1 decoy receptors. PD-1 decoy receptors, fused to the intracellular stimulatory domain, were demonstrated to convert or compete with the immunosuppressive signal and enhance the anti-tumor activity of CAR-T cells in giant cell carcinoma [92]. Zou et al. engineered CAR-T cells to express three immune checkpoint inhibitory receptors, namely, PD-1, Tim-3 and Lag-3, to downregulate their immune checkpoint receptors on CAR-T cells and to form a novel type of CAR-T cell (PTL-CAR-T cells). It was demonstrated that blocking these immune checkpoints can promote infiltration, prolong survival and enhance the anti-tumor activity of CAR-T cells (Figure 3f) [93].

#### 4.2.3. Immunogenicity of CAR-T Cells

The extracellular domain of CARs, especially derived from mouse and other non-human antibodies, has immunogenicity and can induce both humoral and cellular anti- CAR immune responses in solid tumor therapy, which limit the efficacy and cause adverse events and even treatment failure [94]. Several studies have demonstrated the negative effects of immunogenicity of CAR-T cells. Turtle et al. conducted a clinical trial to evaluate CAR-T cells targeting CD19 in treating B cell acute lymphoblastic leukemia (NCT01865617). Although the proliferation of CAR-T cells was observed after the first infusion of defined CD4^+^ and CD8^+^ subsets of CAR-T cells, five patients suffered from persistent leukemia or subsequently relapsed and received a second administration because of anti-CAR transgene immune responses [95]. In a phase 1 trial for the treatment of metastatic colorectal cancer, the immune response to the mouse-derived binding domains of CAR-T cells targeting tumor-associated glycoprotein 72 was reported (11 out of 13 patients) [96]. Moreover, the novel generations of CAR-T cells engineered to express cytokines, suicide genes and immune checkpoint proteins have demonstrated the risks of immunological complications [94].

To eliminate anti-CAR-T cell immune responses, several compounds of CAR-T cells have been designed. As early as 2012, Lanitis et al. designed anti-mesothelin CAR-T cells with pure human scFv to overcome the potential issue of immunogenicity, and reported a significant regression of the tumor size in an ovarian cancer xenograft mouse model after CAR-T cell administration [97]. Lam et al. designed anti-BCMA CAR-T cells with a novel scFv. Unlike the conventional murine scFv consisting of a heavy-chain variable domain and a light-chain variable domain, this new scFv only comprises a humanized heavy-chain variable domain (FHVH33) that can reduce the size of the CAR binding domain and the immunogenicity of CAR-T cells. FHVH33-CAR-T cells presented almost the same cytotoxicity and amounts of cytokines compared to conventional CAR-T cells four or five days after treatment, which indicates that FHVH33 CAR-T cells can be used in clinical applications to avoid the immunogenicity of CAR-T cells [98]. Wagner et al. determined the efficacy of the human-derived CARs mentioned above in reducing the immunogenicity of CAR-T cells. Meanwhile, they also summarized several other methods, including using tumor-specific domains, universal CARs and mutating CAR spacers to reduce anti-CAR immune responses [94].

### 4.3. The Safety of CAR-T Cells in HNSCC

Cytokine profiles in HNSCC are different from hematological malignancies. CD19 was overexpressed in acute lymphoblastic leukemia and chronic lymphocytic leukemia, which was regarded as the promising immunotherapeutic target in hematological malignancies [99]. However, cytokine in HNSCC cells was expressed frequently in other normal tissues. Take HER2 as an example: HER2 is not only expressed in HNSCC but is also expressed in pulmonary tissue; this would cause multi-organ dysfunction in HER2-targeted CAR-T cell therapy of HNSCC [100]. Because of these different cytokine profiles, although the preclinical studies have presented promising results, various adverse events including “on-target, off-tumor” toxicity and CRS have been reported in clinical trials. Furthermore, researchers have proposed corresponding solutions against these adverse events.

#### 4.3.1. On-Target, Off-Tumor” Toxicity in CAR-T Cell Therapy

The low-level expression of tumor-associated antigens in normal tissues is one of the important reasons for the “on-target, off-tumor” toxicity that causes normal tissue damage, which can be life-threatening. Selecting suitable antigens for CAR-T cell therapy and enhancing the selective expression of CARs in the tumor site are of significance in the current research [101]. Kosti et al. designed a stringent hypoxia-sensing CAR-T cell system that expresses pan-ErbB-targeted CARs in the hypoxic site. The results showed that the expression of CAR molecules could be detected on the surface of CAR-T cells only in the hypoxic tumor site of HNSCC, while in normal tissues there were no detectable CAR molecules [102]. Choe et al. designed synthetic notch (synNotch) CAR-T cells for the treatment of glioblastomas. This synNotch receptor can detect tumor-specific antigens such as EGFRvIII expressed on glioblastomas specifically and myelin oligodendrocyte glycoprotein expressed in the central nervous system specifically, and can then induce the expression of tandem CARs targeting ephrin type A receptor 2 (EphA2) and IL13Rα2 as killing targets to achieve the activation of CAR-T cells. In brain slices from a GBM6 tumor-bearing mice model six days after the injection of α-EGFRvIII synNotch–α-EphA2/IL13Rα2 CAR-T cells, primed T cells and apoptosis cells can be detected only in tumor sites [103].

Another way to reduce the “on-target, off-tumor” toxicity of CAR-T cells is to inhibit the function of CAR-T cells, so the concept of inhibitory CAR-T (iCAR-T) cells has been put forward (Figure 3g) [104]. In iCAR-T cells, the intracellular signal domain of traditional CAR-T cells is replaced with PD-1 or cytotoxic T lymphocyte-associated protein 4 (CTLA-4) that can inhibit the proliferation and cytotoxicity of CAR-T cells when CARs recognize the targeting antigens expressed in normal tissues [105].

#### 4.3.2. Cytokine Release Syndrome in CAR-T Cell Therapy

The pathophysiology of CRS is associated with the overexpression of cytokines, including IL-6, TNF-α, IFNγ and IL-1, in a patient’s serum; so, blocking these cytokines can achieve the reversal of adverse events in CAR-T cell therapy against HNSCC [106]. The administration of the STAT pathway inhibitors tocilizumab and itacitinib can play a role in reducing the expression levels of cytokines such as IL-6 and IFNγ in patients or animal models suffering with CRS after anti-CD19 CAR-T cell therapy [107,108]. Moreover, CAR-T cells engineered with suicide genes as a safety switch can also eliminate T cells and inhibit the cytotoxicity effect (Figure 3h). Inducible caspase-9 (*iC9*), as a suicide gene, can achieve conditional dimerization using a chemical inducer of dimerization (CID) and can induce the apoptosis of CAR-T cells [109]. Diaconu et al. designed a CAR-T cell incorporating *iC9* to eliminate CD19-CAR-T cells in vivo. After receiving CID, the expression levels of IFN-γ, IL-6 and TNF-α decreased significantly and no difference in the anti-tumor efficacy was observed compared to receiving the inactive vehicle [110].

Combining CAR-T cell therapy with certain drugs can decrease the CAR-T cytokines’ release. Wei et al. demonstrated that THZ1, the inhibitor of cyclin-dependent kinase 7, has protective effects concerning CRS in CAR-T cell therapy without influencing the anti-tumor effect [111]. Zou et al. showed that the signal transducer and activator of transcription 3 (STAT3) can induce the expression of certain cytokines such as IL-6 and IL-10, and that combining CAR-T cells with STAT3 inhibition can reduce the adverse events of CAR-T cell therapy [112]. As early as 2017, the IL-6Rα/STAT3 pathway inhibitor tocilizumab was approved by the FDA to reduce CRS in CAR-T cell therapy for cancer treatment [113].

## 5. Conclusions

CAR-T cell therapy specifically eliminates tumor cells in an MHC-independent manner and becomes a novel modality for tumor immunotherapy. Remarkable achievements of CAR-T cell therapy for the treatment of hematological malignancies provide researchers with directions and inspirations for CAR-T cell therapy in HNSCC. According to previous studies, CAR-T cells targeting EGFR, HER2, etc., demonstrate anti-tumor efficacy and show promising clinical responses. However, the anti-tumor efficacy and safety of CAR-T cells in treating solid tumors, including HNSCC, still need to be improved. There are still some barriers to be overcome in the application of CAR-T cells for HNSCC treatment:

(1) A lack of suitable tumor-specific targets: Taking EGFR as an example, due to the wide expression of EGFR in normal tissues, CAR-T cells with great affinity for EGFR can cause “on-target, off-tumor” toxicity that can damage the gastrointestinal tract, respiratory system and blood system [114]. Therefore, when selecting targets for HNSCC CAR-T cell therapy, attention should be paid to the specificity of antigen expression to reduce the toxicity to normal tissues.

(2) CRS as one of the common adverse events in CAR-T cell treatment: theoretically speaking, the conversion of CAR-T-cell-induced HNSCC tumor cell pyroptosis into apoptosis can effectively inhibit the occurrence of CRS.

(3) The singleness of drug delivery: Post-surgery drug delivery systems have been reported to reduce recurrence and metastasis and improve the quality of life of patients. However, currently, the intravenous injection of CAR-T cells is still a common method of drug delivery. Therefore, research on CAR-T cells should also focus on the use of CAR-T cell therapy at the surgical site to reduce residual HNSCC.

(4) The personalization of CAR-T cell production for the treatment of HNSCC patients: Due to the efficacy barriers such as the genetic heterogeneity of HNSCC in CAR-T cell therapy, the high cost of synthesis of CAR-T cell productions and long synthesis period make it difficult for patients to receive treatment in time. Thus, research on UniCAR-T cells plays an important role in promoting the applications of CAR-T cells for the clinical treatment of HNSCC.

In summary, although facing many barriers, it is believed that with the development of genetic engineering, drug delivery systems and immunotherapy, CAR-T cells will help to improve the treatment efficacy of HNSCC.

## Authors Contributions

L.-L.B. and B.L. conceived the idea; H.-Q.W. and R.F. performed the literature search and drafted the manuscript; H.-Q.W., G.Y. and Q.-W.M. revised and edited the manuscript; L.-L.B. and B.L. supervised and revised the manuscript. All authors have read and agreed to the published version of the manuscript.

## Figures and Tables

**Table 1:** Research progress of CAR-T cell therapy in head and neck squamous cell carcinomas (HNSCCs).

Target	Introduction	Reference
Preclinical study		
HER2	CD28. CD3-ζ/CAd	[42]
EGFR	-	[43]
CD70	4-1BB.CD3-ζ	[44]
MUCI	4-1BB.CD3-ζ/IL-12	[45]
CD44v6	CD28. CD3-ζ	[46]
B7-H3	-	[47]
CD98 + EGFR	UniCAR-T	[48]
**Clinical trial**		
ERBb2/HER2	NCT01818323 NCT03740256	[49,50]
EpCAM	NCT02915445	
NKG2DL	NCT04107142	
LMP1	NCT02980315	
**Potential target**		
FAP	Overexpression of HNSCC cells compared to adjacent tissue controls (*p* < 0.0005); FAP-targeting CAR-T cells have been used in treating other solid tumors	[44,51,52,53]
HER3	Overexpression of HPV-positive HNSCC cells compared to HPV-negative HNSCC cells (*p* = 0.0007); HER3-targeting CAR-T cells have been used in treating mice bearing breast tumor cells	[54,55]
NKGD2	Overexpression of MICA and MICB in HNSCC cells compared to adjacent tissue controls (*p* < 0.0005); NKGD2-targeting CAR-T cells for treatment of acute myeloid leukemia and multiple myeloma have been used in clinical trial	[44,56]

## Data Availability

Not applicable.

## Abbreviations

HNSCC:	head and neck squamous cell carcinoma;
CAR-T:	chimeric antigen receptor T;
MHC:	non-major histocompatibility complex;
scFv:	single-chain variable fragment;
V_L_:	variable light chain;
V_H_:	variable heavy chain;
TRUCK:	T cells redirected for universal cytokine killing;
IL:	interleukin;
UniCAR-T:	universal CAR-T;
CRS:	cytokine release syndrome;
EGFR:	epidermal growth factor receptor;
GBM:	glioblastoma;
HER2:	human epidermal growth factor receptor 2;
GD2:	ganglioside D2;
FPA:	fibroblast activation protein;
MUC1:	mucin 1;
EpCAM:	epithelial cell adhesion molecule;
NKG2DL:	natural killer group 2 member D ligand;
LMP1:	latent membrane protein;
TME:	tumor microenvironment;
NIR:	near infrared
aPDL1:	anti-programmed death-ligand-1-blocking antibody;
PTT:	photothermal therapy;
CXCR:	C-X-C motif chemokine receptor;
HPSE:	heparinase;
TanCAR-T:	tandem CAR-T cells;
SUPRA:	split, universal and programmable;
OVs:	oncolytic viruses;
CAd:	binary oncolytic adenovirus;
PD-L1:	programmed cell death-ligand 1;
FHVH33:	humanized heavy-chain variable domain;
synNotch:	synthetic notch;
EphA2:	ephrin type A receptor 2;
iCAR-T:	inhibitory CAR-T;
iC9:	caspase-9;
CID:	chemical inducer of dimerization;
STAT3:	signal transducer and activator of transcription 3.
